# Supplementary material for: The Rtf1/Prf1-dependent histone modification axis counteracts multi-drug resistance in fission yeast
Source: Life Sci Alliance. 2024 Mar 21;7(6):e202302494. doi: 10.26508/lsa.202302494 (PMC10958104; doi:10.26508/lsa.202302494)
Supplement: Supplementary file 1 [file LSA-2023-02494_TableS1.docx]

**Table S1. *S. pombe* strains used in this study**.

| **Name** | **Genotype** | **Source** |
| --- | --- | --- |
| JT204 | *h- ade6-M216* | Tanny et al., 2007 |
| JT362 | *h+ leu1-32 ura4-D18 his3-D1 ade6-M210* | Sanso et al 2020 |
| JT98-1 | *h- htb1-K119R::kanMX6 ade6 leu1-32 ura4-D18* | Tanny et al 2007 |
| JT113-2 | *h- prf1∆::hphMX4 ade6* | Mbogning et al., 2013 |
| JT1144 | *h+ rpb3-HA::kanMX6 ura4-D18* | This study |
| JT1050 | *h- rpb1-WT::natMX6 leu1-32 ura4-D18 his3-D1 ade6-M210* (marker placed 3’ of *rpb1^+^*) | Sanchez et al, 2018 |
| JT1051 | *h- rpb1-Y1F::natMX6 leu1-32 ura4-D18 his3-D1 ade6-M210* | Sanchez et al, 2018 |
| JT1052 | *h- rpb1-S2A::natMX6 leu1-32 ura4-D18 his3-D1 ade6-M210* | Sanchez et al, 2018 |
| JT1053 | *h- rpb1-T4A::natMX6 leu1-32 ura4-D18 his3-D1 ade6-M210* | Sanchez et al, 2018 |
| JT1054 | *h- rpb1-S7A::natMX6 leu1-32 ura4-D18 his3-D1 ade6-M210* | Sanchez et al, 2018 |
| JT331 | *h- brl1∆::hphMX4 ade6-M210* | Tanny et al 2007 |
| JT100 | *h+ set1∆::kanMX6 ade6 leu1-32 ura4-D18* | Tanny et al 2007 |
| JT1147 | *h+ rad3::ura4+ ura4-D18 leu1-32* | NBRP FY32725 |
| JT876 | *h- cds1∆::ura4+ leu1-32 ura4-D18* | NBRP FY7448 |
| JT877 | *h- chk1∆::ura4+ ade6-704 ura4-D18 leu1-32* | NBRP FY7811 |
| JT142 | *h- set2∆::hphMX6 ade6* | Sanso et al 2020 |
| JT1164 | *h? rpb1-WT::natMX6 rpb3-HA:kanMX6* | This study |
| JT1165 | *h? rpb1-Y1F::natMX6 rpb3-HA:kanMX6* | This study |
| JT1166 | *h? rpb1-S2A::natMX6 rpb3-HA:kanMX6* | This study |
| JT1167 | *h? rpb1-T4A::natMX6 rpb3-HA:kanMX6* | This study |
| JT1168 | *h? rpb1-S7A::natMX6 rpb3-HA:kanMX6* | This study |
| JT1169 | *h? prf1∆::hphMX4 rpb3-HA:kanMX6* | This study |
| JT1170 | *h? brl1∆::hphMX4 rpb3-HA:kanMX6* | This study |
| JT1196 | *h? htb-K119R::kanMX6 rpb3-HA:kanMX6* | This study |
| JT340 | *h- spt5(7)::ura4+ leu1-32 ura4-D18 his3-D1 ade6-M210* | Schneider et al 2010 |
| JT341 | *h- spt5-T1A(7)::ura4+ leu1-32; ura4-D18 his3-D1 ade6 (7 repeats)* | Schneider et al 2010 |
| JT342 | *h- spt5-T1E(7)::ura4+ leu1-32 ura4-D18 his3-D1 ade6 (7 repeats)* | Schneider et al 2010 |
| JT343 | *h- spt5∆C::ura4+ leu1-32 ura4-D18 his3-D1 ade6* | Schneider et al 2010 |
| JT744 | *h+ mst2∆::natMX6 leu1-32 ura4DS/E ade6-210* | S. Jia |
| JT745 | *h- epe1∆::kanMX4 leu1-32 ura4DS/E ade6-210 mat1Msmt0* | S. Jia |
| JT746 | *h- bdf2∆::natMX6 leu1-32 ura4DS/E ade6-210 mat1Msmt0* | S. Jia |
| JT747 | *h+ nto1∆::kanMX6 leu1-32 ura4DS/E ade6-210* | S. Jia |
| JT306 | *h+ gcn5∆::ura4+ ura4-D18 ade6-M210* | F. Winston |
| JT307 | *h+ spt8∆::ura4+ ura4-D18 ade6-M216* | F. Winston |
| JT308 | *h+ spt20∆::ura4+ ura4-D18 ade6-M216* | F. Winston |
| JT281 | *h- pob3∆::hphMX6* | R. Allshire |
| JT277 | *h- hos2∆::LEU2 leu1-32* | K. Ekwall |
| JT232 | *h+ tfs1∆::natMX6* | A. Ladurner |
| JT84 | *h+ rhp6∆::kanMX6 ade6-M216* | Tanny et al, 2007 |
| JT117 | *h+ hip1∆::ura4+ ade6-M210 leu1-32 ura4-D18* | S. Whitehall |
| JT118 | *h+ slm9∆::ura4+ leu1-32 ura4-D18* | S. Whitehall |
| JT121 | *h+ bre1A∆::hphMX4 ade6-M216* | Tanny et al, 2007 |
| JT122 | *h+ bre1B∆::hphMX4 ade6-M210 ura4+* | Tanny et al, 2007 |
| JT123 | *h+ pht1∆::hphMX4 ade6+* | Tanny et al, 2007 |
| JT621 | *h+ alp13∆::hphMX6* | Sanso et al 2020 |
| JT622 | *h+ cph1∆::hphMX6* | Sanso et al 2020 |
| JT623 | *h+ cph2∆::hphMX6* | Sanso et al 2020 |
| JT261 | *h+ lsk1∆::ura4+ leu1-32; ura4-D18* | Mbogning et al, 2015 |
| JT301 | *h? swd2.2∆::kanMX4* | Bioneer |
| JT893 | *h+ ppn1∆::hphMX4* | V. Vanoosthuyse |
| JT409 | *h+ paf1∆::kanMX4 ade6-M216 ura4-D18 leu1-32* | Mbogning et al, 2013 |
| JT410 | *h+ leo1∆::kanMX4 ade6-M216 ura4-D18 leu1-32* | Mbogning et al, 2013 |
| JT411 | *h+ tpr1∆::kanMX4 ade6-M216 ura4-D18 leu1-32* | Mbogning et al, 2013 |
| JT412 | *h+ cdc73∆::kanMX4 ade6-M216 ura4-D18 leu1-32* | Mbogning et al, 2013 |
| JT1047 | *h- spt5-WT::ura4+ ura4-D18 (18 repeat version)* | MacKinnon et al, 2023 |
| JT1048 | *h- spt5-T1A::ura4+ ura4-D18 (18 repeat version)* | MacKinnon et al, 2023 |
| JT1049 | *h- spt5-T1E::ura4+ ura4-D18 (18 repeat version)* | MacKinnon et al, 2023 |
| JT560 | *h- H4.2ura4 tag H3.1/H4.1::his3+H3.3/H4.3::arg3+ his3-D1 ura4-D18 leu1-32 ade6-210 arg3-D4 ade6-otr* | Xhemalce 2010 |
| JT561 | *h- H3.1/H4.1::his3+H3.3/H4.3::arg3+H3.2K4R his3-D1 ura4-D18 leu1-32 ade6-210 arg3-D4 ade6-otr* | Xhemalce 2010 |
| JT573 | *h- hrp1∆::ura4 ade6-M210 leu1-32 ura4-D18* | K. Ekwall |
| JT297 | *h- ubp8∆::hphMX6 ade6-M216* | Tanny et al 2007 |
| JT1261-1 | *h- spd1∆::ura4+ ura4-D18 leu1-32 ade6-704* | NBRP FY3432 |
| JT1330 | *h? spd1∆::ura4+ htb-K119R::kanMX6; ura4-D18* | This study |
| JT1331 | *h? spd1∆::ura4+ set1∆::kanMX6 ura4-D18* | This study |
| JT1338 | *h? spd1∆::ura4+ cds1∆::ura4+ ura4-D18* | This study |
| JT1334 | *h? spd1∆::ura4+ rpb1-WT::natMX6 ura4-D18* | This study |
| JT1335 | *h? spd1∆::ura4+ rpb1-Y1F::natMX6 ura4-D18* | This study |
| JT1336 | *h? spd1∆::ura4+ rpb1-T4A::natMX6 ura4-D18* | This study |
| JT1337 | *h? spd1∆:ura4+ rpb1-S7A::natMX6 ura4-D18* | This study |
| JT1257 | *h- rhp18∆::ura4+ ura4-D18 leu1-32 ade6-704* | NBRP FY3123 |
| JT1278 | *h? rhp18∆::ura4+ htb-K119R::kanMX6 ura4-D18* | This study |
| JT1279 | *h? rhp18∆::ura4+ set1∆::kanMX6 ura4-D18* | This study |
| JT1280 | *h? rhp18∆::ura4+ cds1::ura4+ ura4-D18* | This study |
| JT1281 | *h? rhp18∆::ura4+ set2∆::hphMX6 ura4-D18* | This study |
| JT1294 | *h? rhp18∆::ura4+ rpb1-WT::natMX6 ura4-D18* | This study |
| JT1295 | *h? rhp18∆::ura4+ rpb1-Y1F::natMX6 ura4-D18* | This study |
| JT1296 | *h? rhp18∆::ura4+ rpb1-T4A::natMX6 ura4-D18* | This study |
| JT1297 | *h? rhp18∆::ura4+ rpb1-S7A::natMX6 ura4-D18* | This study |
| JT1153 | *h? rad3::ura4+ set1∆::kanMX6 ura4-D18* | This study |
| JT1157 | *h? rad3::ura4+ htb1-K119R::kanMX6 ura4-D18* | This study |
| JT1236 | *h? rad3::ura4+ ura4-D18 rpb1-WT::natMX6* | This study |
| JT1266 | *h? rad3::ura4+ ura4-D18 rpb1-Y1F::natMX6* | This study |
| JT1237 | *h? rad3::ura4+ ura4-D18 rpb1-T4A::natMX6* | This study |
| JT1238 | *h? rad3::ura4+ ura4-D18 rpb1-S7A::natMX6* | This study |
| JT884 | *h? cds1::ura4+ htb1-K119R::kanMX6 ura4-D18* | This study |
| JT885 | *h? chk1::ura4 htb1-K119R::kanMX6 ura4-D18* | This study |
| JT1212 | *h? set1∆::kanMX6 cds1::ura4+ ura4-D18* | This study |
| JT1213 | *h? set1∆::kanMX6 chk1::ura4+ ura4-D18* | This study |
| JT1267 | *h? cds1::ura4+ ura4-D18 rpb1-WT::natMX6* | This study |
| JT1275 | *h? cds1::ura4+ ura4-D18 rpb1-Y1F::natMX6* | This study |
| JT1319 | *h? cds1::ura4+ ura4-D18 rpb1-T4A::natMX6* | This study |
| JT1276 | *h? cds1::ura4+ ura4-D18 rpb1-S7A::natMX6* | This study |
| JT1303 | *h? chk1::ura4+ ura4-D18 rpb1-WT::natMX6* | This study |
| JT1304 | *h? chk1::ura4+ ura4-D18 rpb1-Y1F::natMX6* | This study |
| JT1305 | *h? chk1::ura4+ ura4-D18 rpb1-T4A::natMX6* | This study |
| JT1306 | *h? chk1::ura4+ ura4-D18 rpb1-S7A::natMX6* | This study |
| JT1121 | *CFP-suc22 ade6-704 leu1-32 ura4D18 h-* | 1. Carr |
| JT1120 | *cdc22-yECitrine::kanMX6 ade6-704 leu1-32 ura4D18 h+* | 1. Carr |
| JT1353 | *cdc22-yECitrine::kanMX6 htb1-K119R::kanMX6 h?* | This study |
| JT1354 | *CFP-suc22 set1∆::kanMX6 h?* | This study |
| JT1355 | *CFP-suc22 htb1-K119R::kanMX6 h?* | This study |
| JT1385 | *pap1∆::ura4 ura4-D18 h-* | NBRP FY19147 |
| JT1395 | *pap1∆::ura4 htb1-K119R::kanMX6 ura4-D18 h?* | This study |
| JT1396 | *pap1∆::ura4 set1∆::kanMX6 ura4-D18 h?* | This study |
